# The Role of Hypothalamic Inflammation in Diet-Induced Obesity and Its Association with Cognitive and Mood Disorders

**DOI:** 10.3390/nu13020498

**Published:** 2021-02-03

**Authors:** Sofia Dionysopoulou, Evangelia Charmandari, Alexandra Bargiota, Nikolaos F Vlahos, George Mastorakos, Georgios Valsamakis

**Affiliations:** 1 Division of Endocrinology, Metabolism and Diabetes, Hippocratio General Hospital, 11527 Athens, Greece; sofiadion88@gmail.com; 2 Division of Endocrinology, Metabolism and Diabetes, First Department of Pediatrics, National and Kapodistrian University of Athens Medical School, ‘Aghia Sophia’ Children’s Hospital, 11527 Athens, Greece; evangelia.charmandari@googlemail.com; 3 Division of Endocrinology and Metabolism, Center for Clinical, Experimental Surgery and Translational Research, Biomedical Research Foundation of the Academy of Athens, 11527 Athens, Greece; 4 Department of Endocrinology and Metabolic Diseases, University Hospital of Larisa, Medical School of Larisa, University of Thessaly, 41334 Larisa, Greece; abargio@yahoo.gr; 5 2nd Department of Obstetrics and Gynecology, Areteion University Hospital, Medical School, National and Kapodistrian University of Athens, 11528 Athens, Greece; nfvlahos@gmail.com; 6 Endocrine Unit, Areteion University Hospital, Medical School, National and Kapodistrian University of Athens, 11528 Athens, Greece; mastorakg@gmail.com

**Keywords:** diet, obesity, hypothalamic inflammation, cognitive disorders, mood disorders, depression, dementia

## Abstract

Obesity is often associated with cognitive and mood disorders. Recent evidence suggests that obesity may cause hypothalamic inflammation. Our aim was to investigate the hypothesis that there is a causal link between obesity-induced hypothalamic inflammation and cognitive and mood disorders. Inflammation may influence hypothalamic inter-connections with regions important for cognition and mood, while it may cause dysregulation of the Hypothalamic-Pituitary-Adrenal (HPA) axis and influence monoaminergic systems. Exercise, healthy diet, and glucagon-like peptide receptor agonists, which can reduce hypothalamic inflammation in obese models, could improve the deleterious effects on cognition and mood.

## 1. Introduction

The prevalence of obesity has been increasing during the last decades [1]. In 2016, approximately 39% of adults older than 18 years were characterized as overweight, and about 13% were obese [1]. A significant part of the population in developed countries has unhealthy dietary habits, consuming high-fat and high glycemic index products [2]. It is well established that this kind of diet can lead to increased body weight and obesity, and that it is associated with metabolic disorders such as insulin resistance, type 2 diabetes mellitus (T2DM), and dyslipidemia. The excess fat in obese individuals is stored mainly in subcutaneous adipose tissue, but also in visceral adipose tissue [3,4] and non-adipose tissue organs, such as liver, pancreas, skeletal muscles, and vessels [5,6,7]. The fat stored in non-adipose tissue organs is characterized as ectopic fat. In addition, obesity is characterized by low-grade chronic inflammation in adipose tissue [8] and is associated with inflammation in other tissues. The increase of immune cell infiltration and proinflammatory activation in intramuscular and perimuscular adipose tissue in obese subjects reflect the inflammation of skeletal muscles [9]. In addition, pancreatic islet cell inflammation has been described in obese and T2DM individuals in rodent and human studies [10]. In addition, obesity promotes liver inflammation by enhancing secretion of interleukin (IL) 6 and tumor necrosis factor (TNF) [11].

However, obesity is associated not only with peripheral-tissue inflammation, but also with neuroinflammation, such as hypothalamic inflammation, as part of the ectopic fat deposition. According to research in rodent models, markers of hypothalamic inflammation are present as early as 1 to 3 days after high-fat diet consumption, indicating that hypothalamic inflammation occurs even before the onset of weight gain [12]. In addition, hypothalamic inflammation occurs in patients with obesity and T2DM, and may play a role in the pathophysiology of these diseases [13]. The hypothalamus is a neuroendocrine organ which integrates neuronal and hormonal stimuli and regulates endocrine functions via numerous and complicated connections with other brain regions and the periphery. Obesity-induced neuroinflammation affects not only hypothalamus, but also other brain regions important for cognition and mood, such as hippocampus. Activated microglia were found in hippocampus of mice fed with high-fat diet for 20 weeks [14]. The inflammatory process has been suggested to be the link between obesity and central and peripheral nervous disorders, including cognitive and mood disorders [15].

Cognition includes intellectual functions as attention, consolidation of knowledge, memory, comprehension of language, evaluation, and decision making. Cognitive disorders refer to a group of symptoms which are caused by deficits of the aforementioned functions. The most common and obvious of these deficits concern memory disorders. The term mood disorders include all types of depression and bipolar disorders [16]. Cognitive disorders such as dementia, and mood disorders such as depression, have important physical, psychological, social, and financial implications, especially for older people.

Dementia is a common disease, with an estimated prevalence of 5–8% in the general population aged over 60 years [17]. Early onset dementia (defined as the onset of symptoms before the age of 65 years) accounts for up to 9% of cases. The major forms of dementia are Alzheimer’s disease (AD), vascular brain injury (VBI), Lewy Bodies disease (LBD), and a group of diseases that contribute to frontotemporal dementia [17]. Alzheimer’s disease is the most common form, since it contributes towards 60–70% of the cases. Alzheimer’s disease, VBI, and LBD often coexist [18,19,20,21]. The most relevant risk factor for all types of dementia is aging [17]. Family history, APOE4 genotype, depression, hypertension, vascular factors, dyslipidemia, obesity, DM, insulin resistance, and traumatic brain injury have also been established as risk factors for AD [22,23,24,25]. Vascular dementia has many common risk factors with peripheral vascular disease [26].

Depression is considered the major cause of morbidity worldwide [27], while its prevalence differs among different countries and ethnic groups (e.g., 3% in Japan, 17% in USA) [28]. Depression is a multifactorial disease, as it is associated with genetics, social and financial status, stressful life events, comorbidity with medical illnesses, and neurodegenerative diseases [28,29,30,31]. Both dementia and depression can impose significant psychological, physical, and financial burden to patients and caregivers and cost to governments a significant percentage (0.2–1.4%) of the gross domestic product [27].

Recent studies have associated high-fat diet with alterations in central nervous system, such as neuroinflammation, decreased hippocampal neurogenesis, and changes in mitochondrial function [32,33].

The aim of this review is to summarize the evidence regarding the possible physiopathologic relationship between diet- and/or obesity-induced hypothalamic inflammation with cognitive and mood disorders. We focus on hypothalamic inflammation, as most of the available data concerning obesity-induced neuroinflammation refer to the impact of obesity in this specific area.

## 2. Methods

For this review, the Medline/PubMed database was researched until May 2020 for studies published in English in the last decade (2010–2020) with the search terms “diet”, “metabolic syndrome”, “obesity”, and cross-referencing them with the terms “depression/mood disorders”, “dementia”, “Alzheimer disease”, “cognitive disorders” concurrently with the term “hypothalamic inflammation”. The results of this search include reviews, animal studies, human cohort, and cross-sectional studies, but randomized control trials (RCTs) on the topic were not found.

## 3. Mechanisms of Hypothalamic Inflammation and the Effects of Diet and Obesity

### 3.1. Mechanisms Involved in Hypothalamic Inflammation

As mentioned above, it is well established that diet-induced obesity is associated with hypothalamic inflammation. Studies in rodents have shown that markers of inflammation are increased after a high-fat diet. Moreover, hypothalamic inflammation has been detected within 24 to 72 h of high-fat diet in mice, indicating that hypothalamic inflammation precedes the onset of obesity. High-fat diet triggers an inflammatory response in the hypothalamus of rats [34]. As far as pathology is concerned, hypothalamic inflammation is characterized by glial cell proliferation, including infiltration of microglia and proliferation of astrocytes [35,36]. Many methods have been used to detect hypothalamic inflammation. The quantification of mRNA that encodes inflammatory mediators is an indicator of inflammation. Moreover, hypothalamic microglia-specific and astrocytes-specific gene expression indicates the activation and proliferation of microglia and astrocytes that characterize hypothalamic inflammation. In addition, the immunohistochemical detection of microglia markers in hypothalamic tissue documents hypothalamic inflammation [12]. Hyperintensity in T2 in brain magnetic resonance imaging (MRI) has been used as a measure of inflammation in humans [35].

A lot of research has been done, investigating the mechanisms underlying hypothalamic inflammation such as the Toll-like receptors 4 (TLR-4) pathway. The activation of TLR-4 further activates the IKB kinase/nuclear factor κB (NF-κB) pathway, forcing the transcription of proinflammatory cytokines such as IL-6, IL-8, TNF, IL1β [37,38,39]. Moreover, this pathway promotes the inflammatory response in microglia cells, which are the resident macrophage cells of hypothalamus [39]. In addition, TLR-4 activation activates JNK, which, in its turn promotes the activation of endoplasmic reticulum and stabilizes the mRNA of inflammatory cytokines and other inflammatory mediators. JNK can be activated by many inflammatory cytokines and environmental factors [37,40,41]. The activation of these pathways leads to increased cytokines, such as interleukin (IL)-1β, interleukin (IL)-6, tumor necrosis factor (TNF)-a, chemokins, and other proinflammatory factors [42].

Endoplasmic reticulum (ER) is an organelle in eukaryotic cells which regulates protein folding and other steps of protein synthesis [41,43]. The ER stress response is an adaptive mechanism resulting to adjustment of protein synthesis in relation to changes of cell homeostasis, such as those resulting from inflammation [44,45]. At the early stage of this response, the adaptive mechanism called unfolded protein response maintains cellular homeostasis [43]. When ER stress response is prolonged, the unfolded protein response fails, and an apoptotic response ensues [46]. Inflammation and ER stress response enhance each other and cause dysfunction of leptin signaling pathway in the hypothalamus of obese mice [47].

The blood-brain barrier prevents pathogens from crossing into the central nervous system [48]. The hypothalamic arcuate nucleus, as a circumventricular organ, lacks a typical blood-brain barrier, while it is responsible for the control of eating and energy expenditure. It is composed of two neuronal subpopulations, the orexigenic agouti-related peptide (AgRP)/neuropeptide Y (NPY) neurons and anorexigenic proopiomelanocortin (POMC)/cocaine and amphetamine regulated transcript (CART) neurons [49]. Chronic high-fat diet leads to the loss of POMC/CART neurons [12,37]. Thus, chronic high-fat diet may cause hypothalamic inflammation which alters neuronal control of appetite, caloric intake, and energy expenditure, causing a vicious circle of obesity.

On the other hand, autophagy is a protective mechanism which maintains cellular homeostasis and allows adaptation to environmental conditions. In case of intracellular stress, such as organelle dysfunction, autophagy is activated in order to maintain cellular homeostasis. Autophagy is active in the hypothalamus of normal mice, whereas malfunctioning autophagy can lead to hypothalamic inflammation [50].

### 3.2. Effects of Diet and Obesity on the Inflammatory Pathways

Diet and obesity can affect the aforementioned mechanisms of hypothalamic inflammation. Saturated fatty acids activate TLR-4 to initiate inflammatory signaling in astrocytes, microglia, and neurons [37,51,52,53]. Astrocytes are also activated by high-fat diet [12], while astrocytosis can stimulate the NFκB pathway through the TLR-4 pathway [54,55]. The saturated fatty acids-induced activation of TLR-4 triggers this ER stress response [41]. ERS response can be also induced by hyperglycemia and hyperlipidemia [56]. High-fat diet can induce ERS in hypothalamus, promoting inflammation [40].

In addition, Western diet (high-fat, high-glycemic index) can increase the permeability of blood-brain barrier [57]. As mentioned above, obesity is characterized by low grade inflammation and increased circulation of inflammatory cytokines. Thus, the hypothalamus is sensitive to circulating inflammation markers, such as the cytokines IL-1β and TNF, which can initiate central inflammation [48,58]. Moreover, autophagy appears defective or disrupted when associated either with high-fat diet-induced obesity [12,50] or with hypothalamic inflammation [39,59,60], respectively. Thus, defect of hypothalamic autophagy under conditions of high-fat diet may be one of the mechanisms which promote hypothalamic inflammation [50,61].

The proposed mechanisms leading to obesity-induced hypothalamic inflammation discussed above are presented in Table 1.

## 4. Association of Diet and Obesity with Cognitive and Mood Disorders

### 4.1. Association of Diet and Obesity with Cognitive Disorders

Obesity and cognitive impairment often coexist, especially among older patients. A large number of studies have investigated whether there is an association and a causal relationship between obesity and cognitive impairment. Some studies have shown conflicting results, revealing the complexity of the association of these morbidities. An observational study suggests that abdominal obesity increases the risk of cognitive impairment later in life. In that study, the results were adjusted for age, education level, hypercholesterolemia, hypertension, and diabetes [60,62]. Interestingly, a 3-year prospective study suggests that older women who were overweight or obese at baseline and their weight remained stable had lower risk for developing cognitive impairment than other weight and sex groups [61,63]. Adolescents with metabolic syndrome showed lower cognitive performance compared with their normal weight counterparts [62,64], indicating a link between obesity and brain function. A recent meta-analysis suggests that obesity increases the risk of vascular dementia, but it is not associated with all-type dementia [63,65]. Interestingly, in the same study, the risk of all-cause dementia is found increased in overweight patients. According to another meta-analysis, being obese in mid-life is positively correlated with the risk of later dementia, but obesity in late life is negatively correlated with dementia [64,66]. In a meta-analysis, the intentional weight loss was shown to improve memory and executive/attention functioning in obese, but not overweight, individuals [65,67].

As far as diet is concerned, saturated fatty acids enhance the risk of cognitive impairment (specifically in AD), however, no association has been found between total fats and cognitive outcomes. Moreover, mono- and poly- unsaturated fats are not associated with the risk of cognitive decline [66,68]. Elsewhere, mono-unsaturated fatty acids have been proposed to play a protective role against cognitive decline [67,69].

Furthermore, diet is associated with cognitive dysfunction in animal studies. Juvenile exposure to a high-fat diet can impair spatial memory in mice [70], while acute exposure in high-fat diet can affect long-term hippocampal-dependent memory and hippocampal plasticity in juvenile male rats [71]. Moreover, working memory and reference memory were impaired in minipigs fed with high-fat/low-carbohydrates and a low fat/high-sucrose diet, compared with minipigs fed with the standard low-fat/low-sugar/high-carbohydrates diet [72]. In line with these results, minipigs fed with a Western diet (high-fat/high-sugar) showed impaired working memory [73]

### 4.2. Association of Diet and Obesity with Mood Disorders

Obesity and depression are both common morbidities that influence the quality of life and often occur concomitantly at the same patients. Several studies have investigated the relationship between these two conditions. According to recent studies, there is a positive association between obesity and depression, since obesity increases the risk of depression, a trend that seems to be greater among female patients [74,75,76,77,78]. Furthermore, obese patients with metabolically unhealthy profile have higher risk of depression, compared with obese ones with metabolically healthy profile [79]. Obesity and depression often share common lifestyle factors, such as smoking and decreased activity. Nevertheless, these factors do not significantly explain the co-occurrence of these two morbidities [75,80]. In addition, antidepressant drugs do not have a significant impact on weight, while depression itself, rather than anti-depressant therapy, increases the risk of obesity [81,82,83]. In line with these results, in women, research has revealed a bi-directional relationship between obesity and depression, with a stronger association to the direction from depression to obesity [80].

Similarly, an animal study supports that a diet rich in saturated fat and sucrose induced anxiety-like behavior in rats, whereas a diet rich in olive oil fat and sucrose did not [84]. Another animal study showed that a high-fat diet during 16 weeks can cause anxiety and anhedonia in rats and can affect synaptic plasticity [85]. Moreover, a high-fat diet seemed to induce depressive-like symptoms, such as reduced sociability and sucrose preference, in mice [86]. Dietary limitations seemed to reverse obesity-induced anhedonia in rats [87]. In addition, Western diet increases anxiety-like behavior and progressively depressive-like behavior in mice, while it induces the expression of proinflammatory cytokines in the hypothalamus and hippocampus [88]. In addition, minipigs fed with Western diet showed anxiety-like behavior and lack of motivation towards rewards, while the motivation seemed to be recovered after weight loss interventions [73]. Interestingly, maternal Western diet during gestation and lactation seemed to increase stress level in the adult, lean descendants [89].

### 4.3. Common Pathophysiologic Mechanisms Associating Obesity with Cognitive and Mood Disorders

Many mechanisms have been proposed as the link between obesity and either cognitive or mood disorders. Firstly, obesity is believed to influence brain structure. Midlife obesity is associated with brain atrophy [90]. In addition, obesity is associated with hippocampal and frontal lobe atrophy, myelin and neuronal dysfunction in the frontal lobe, and decreased gray matter volume [91,92,93]. In line with these results, western diet is linked with smaller hippocampal volume [94]. As hippocampus plays a central role in mood and cognition and frontal lobe is crucial for executive functions [95], obesity-induced structural alteration of these brain areas has been proposed to cause cognitive decline and to influence mood. In animals, high-fat diet can cause endothelial dysfunction in cerebral vessels [96] and impaired communication between neurons and vessels [97]. These dysfunctions have been associated with cognitive impairment [98]. Moreover, prolonged high-fat diet can induce β-amyloid depositions in rodents’ brains, contributing to AD [99]. In rats, a diet rich in saturated fats can repress hypothalamic PKA signaling, thus inducing depression-like behavior [100].

It is known that one of the metabolic abnormalities that can be caused by obesity is insulin resistance. Recent studies provide evidence that insulin resistance is associated with cognitive and mood disorders. More specifically, research results support that insulin resistance is negatively correlated with hippocampal volume and cognitive performance in middle-aged women at risk of AD [101]. Moreover, a recent meta-analysis supported the association between insulin resistance and depression [102]. The inactivation of the insulin receptor in hypothalamus can induce depressive-like symptoms in rats [103,104]. Leptin resistance is another metabolic dysfunction in obesity [105]. Leptin resistance has been associated with the atypical subtype of major depression [106]. Indeed, in chronic stress conditions, such as depression, the expression of leptin receptor mRNA in the hypothalamus is decreased, and leptin does not inhibit Hypothalamic-Pituitary-Adrenal (HPA) axis as it happens in acute stress situations [107]. In animal studies, chronic stress induces HPA axis hyperactivity and reduced leptin receptor mRNA expression in the hypothalamus [107,108]. In addition, cognitive impairment and depressive and anxiety-like behavior have been noticed in obese rats, chronically exposed to stress. Down-regulation of leptin receptors in the hypothalamus and the hippocampus of these rats has been proposed as a possible cause of these symptoms. [107,109]. Further research is needed to confirm a causal link between obesity-induced leptin dysregulation and depression.

The gut-brain axis is also believed to be a link between obesity and neuropsychiatric disorders. Animal data show that the Western (high-fat, high glycemic index) diet changes the composition of gut microbiome and synaptic plasticity [110]. Moreover, a high sucrose diet can alter gut microbiome and at the same time impair cognitive flexibility along with short term and long term memory [111]. In addition, Bruce-Keller et al. transplanted microbiota from high-fat diet fed mice to lean mice and observed cognitive and behavioral dysfunction and increased neuroinflammation compared to the control group [112]. In line with these results, gut dysbiosis has been proposed to play a role in the pathogenesis of AD [113,114]. Gut-microbiota dysbiosis increases intestinal and blood brain barrier permeability and may contribute in that way to the pathogenesis of neurodegenerative disorders [112,114]. Amyloids and lipopolysaccharides produced by gut bacteria can activate cytokines cascade, inducing the production of proinflammatory cytokines associated with the pathogenesis of AD [114]. The results of a recent RCT, where intervention with probiotic supplements improved the mini-mental test results in AD patients, support the hypothesis that gut-microbiome can contribute to the pathogenesis of this disease [115]. Both obesity and depression are associated with decreased microbiota diversity [116,117,118]. According to animal studies, fecal microbiota transplantation from patients with major depression to germ-free rats can cause depression-like behavior such as anhedonia and anxiety [117,119]. Interestingly, the use of probiotics improved the depression scale score [120], thus providing evidence for the determinant role of gut microbiome in the pathogenesis of depression.

**Figure 1:**
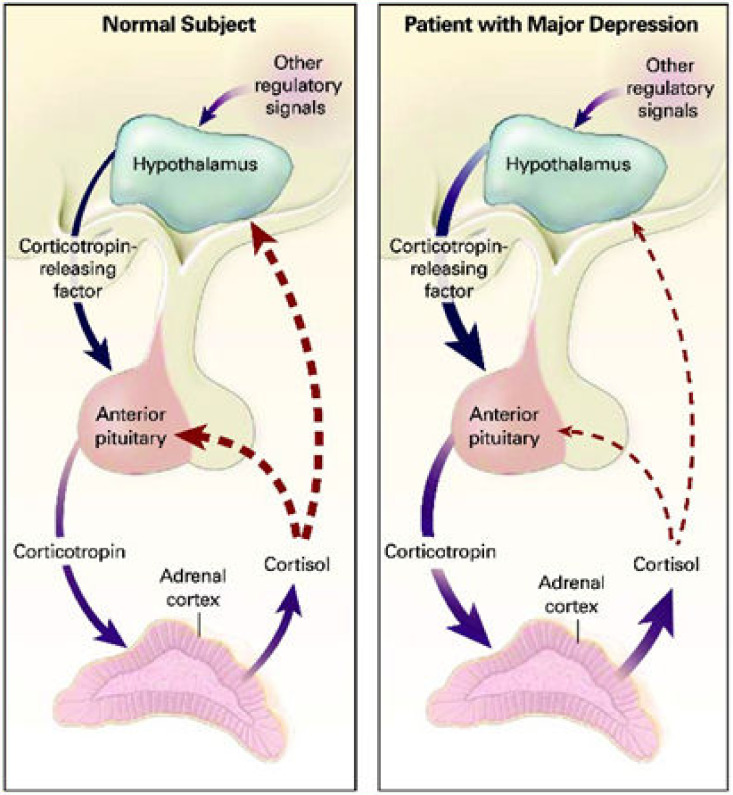
Daskalakis, N.P.; McGill, M.A.; Lehrner, A.; Yehuda, R. (2015). Endocrine Aspects of PTSD: Hypothalamic-Pituitary-Adrenal (HPA) Axis and Beyond. Comprehensive Guide to Post-Traumatic Stress Disorder, 1–14. doi:10.1007/978-3-319-08613-2_130-1 [133] HPA axis dysfunction in patients with major depression. Disrupted HPA axis negative feedback, leads to high cortisol levels in depressed patients.

The dysfunction of HPA axis has been proposed as a common mechanism between obesity and cognitive and mood disorders. In obese individuals, the HPA axis is altered and higher cortisol concentrations are found in adipose tissue [121]. According to a recent meta-analysis, hair cortisol level is positively associated with anthropometric features such as BMI and waist-to-hip ratio [122]. It is known that cortisol is a stress hormone and there is evidence that HPA axis activation is an important factor for mood and cognitive disorders. More specifically, high cortisol concentration has been found in patients with major depression, psychotic and non-psychotic, compared to healthy controls [123]. Additionally, changes in the function of glucocorticoid receptors (GRs) type II (which bind glucocorticoids) that disrupt the HPA axis negative feedback are responsible for HPA axis dysregulation and high cortisol concentrations in depressed patients [124,125,126]. The dysfunction of HPA axis in patients with major depression is presented in Figure 1. In line with these, it has been noticed that many patients with major depression are not capable of reducing cortisol levels after dexamethasone administration, whereas after successful antidepressant therapy, there is a normal reaction to the dexamethasone suppression test [127,128]. At the same time, increased concentration of glucocorticoids can cause neurotoxicity and atrophy in the hippocampus, which plays a vital role in memory and mood regulation [129]. Moreover, elevated cortisol concentrations have been associated with cognitive impairment, no-dementia, and dementia [130]. Interestingly, recent studies show that chronic maternal stress during pregnancy is correlated with elevated cortisol levels in the fetus [131]. It has been proposed that maternal exposure to stress is associated with neuroendocrine and neuroanatomical alterations in the offspring, that can put the offspring into greater risk for behavioral, psychological, and cognitive problems [132].

Inflammation, which is an important pathophysiological link between obesity and mood and cognitive disorders, will be discussed below.

## 5. Presumed Role of Hypothalamic Inflammation in Cognitive and Mood Disorders

Inflammation has been proposed to be an important link between diet-induced obesity and cognitive disorders. There is evidence that diet-induced obesity can cause hypothalamic inflammation. It has been suggested that inflammation influences hypothalamic signaling to other brain regions [58]. As mentioned above, hypothalamic inflammation is detected within days in rats eating a high-fat diet [12], whereas in the same study, inflammation was not found in extra hypothalamic regions. Moreover, other animal studies showed that inflammation markers were increased after high-fat diet specifically in hypothalamus and not in other brain regions [34,44]. It has been suggested that inflammation influences hypothalamic signaling to other brain regions [58]. Research results have shown activation of microglia in the hippocampus, after 20 weeks of high-fat diet [14], elevated markers of astrocytes and microglia in the frontal cortex in mice fed with high fat diet for 14 weeks [134]. Because of the difference in the duration of high-fat diet needed to induce inflammation in different brain regions, it has been hypothesized that obesity-induced inflammation in hypothalamus may proceed of the appearance of inflammation in extra-hypothalamic areas important for cognition and mood [58]. In addition, animal studies suggest that high-fat diet induces neuronal apoptosis in the hypothalamus and the hippocampus [135,136]. In addition, a high-fat diet in juvenile rats may increase expression of IL-1β and TNF in the hippocampus while it is associated with impairment of spatial memory and decreased hippocampal neurogenesis [70]. In a mouse model of metabolic syndrome, Dinel et al. proposed an association of anxiety-like behavior and spatial memory impairment with increased proinflammatory cytokines and reduction of the expression of brain-derived neurotrophic factor (BDNF) in the hippocampus [137]. Furthermore, in recent years, research results support that T-helper (Th) cells, which are characterized by the production of IL-17 (Th 17 cells), are associated with depression [138]. Research results support that obesity induces Th17 cells differentiation [139]. Th17 cells and IL-17A have been found elevated in blood of depressed patients [140,141,142]. Also, Th 17 cells have been proposed to induce neuroinflammation and neuronal death in Alzheimer’s disease models [143]. Of note, it has been reported that interferon (IFN)-α therapy in patients with melanoma or hepatitis C induces depressive symptoms [144,145]. Concentrations of TNF and IL-6 are elevated in depressed individuals compared to healthy ones [146], while CRP concentrations have been associated with depression scale scores and explained 20% of the obesity-related alteration in depression scores at follow-up [147]. These results support the hypothesis that inflammation contributes to the pathogenesis of depression and may be a link between depression and obesity.

Obesity is associated with inflammation with elevated peripheral proinflammatory cytokines which can stimulate HPA axis activity [148,149]. Certain cytokines, such as IL-1β, can cross the blood-brain barrier and affect the central nervous system [48,150]. For example, IL-1 and IL-6 act on receptors in the hypothalamic arcuate nucleus and can influence the expression of NPY [151]. The presence of IL-1β in the hypothalamus during inflammatory conditions can trigger CRH secretion, activating the HPA axis [152]. Human studies have shown that administration of recombinant IL-6 activates the HPA axis, resulting in remarkably increased ACTH, cortisol, and vasopressin (another HPA axis secretagogue) concentrations [148,149]. Experimental results support that exposure to IFN-α can also activate the HPA axis, while administration of IL-1 can increase CRH, ACTH, and glucocorticoids [149,153]. In addition, cytokines, by affecting glucocorticoid receptors (GR), can cause glucocorticoid resistance, restraining the negative feedback of the HPA axis [153,154,155,156]. More specifically, IL-1 induces the expression of isoform beta of GR type II (a dominant negative receptor for GRa-induced transcriptional activity) over the expression of isoform alpha (which binds glucocorticoids and is activated by them) [155]. The former impairs the function of the latter. In addition, IL-1a restrains the function of GR type II by activating p38 mitogen-activated protein kinase [157]. Chronic exposure to IL-1β can alter GR type II signaling as well [156]. These data indicate that glucocorticoid resistance caused by cytokines induces hyper activation of the HPA axis resulting to increased circulating cortisol concentrations. Taking into consideration the aforementioned association between HPA axis dysregulation and neuropsychiatric disorders, it can be hypothesized that an inflammatory state can impact mood and cognition through this HPA axis dysregulation.

**Figure 2:**
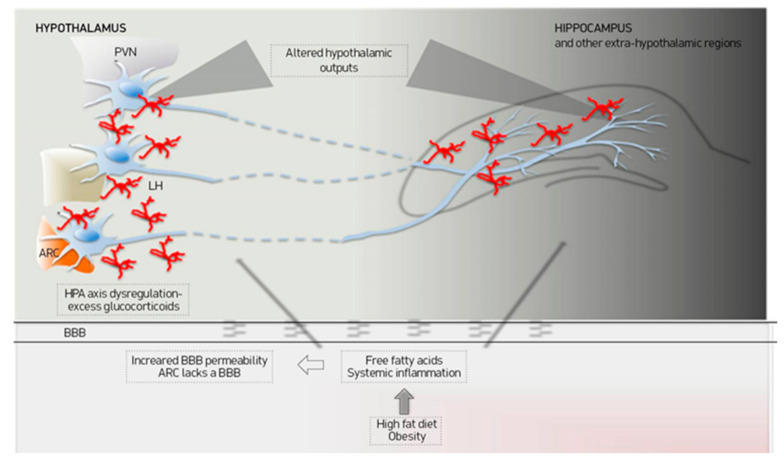
A.A. Miller, S.J. Spencer / Brain, Behavior, and Immunity 42 (2014) 10–21 [58]. Proposed mechanisms of cognitive impairment in obesity. Obesity is characterized by increased proinflammatory cytokines and free fatty acids that can access central nervous system through increased BBB permeability or through ARC that lacks an effective BBB. Hypothalamic inflammation can probably affect the hypothalamic outputs to the other brain regions and the Hypothalamic-Pituitary-Adrenal (HPA) axis activity. ARC: Arcuate nucleus, BBB: Blood-brain barrier, LH: Lateral hypothalamus, PVN: Paraventricular nucleus.

As a key brain site for many biological systems, the hypothalamus has numerous and complicated connections with other brain regions contributing to the regulation of physiological functions including the control of emotion and learning. More specifically, it has been proposed that the hypothalamus communicates directly with the subgenual cortex, which is considered to be implicated in the feeling of sadness [158,159]. The presence of GRs type II in this region and the response to exogenous corticosteroids indicate that the hypothalamus may indirectly influence the subgenual region via HPA axis activation [160,161]. According to a recent study, decreased functional connectivity between the hypothalamus and subgenual cortex is observed in patients with psychotic major depression and this might implicate HPA dysfunction [162]. Moreover, the arcuate nucleus is susceptible to high-fat diet-induced inflammation. It is involved with appetite and satiety regulation via interconnections with other hypothalamic (such as the paraventricular nucleus) and extra hypothalamic regions [163]. Additionally, the arcuate nucleus and the lateral hypothalamus get input signals from regions related to reward, memory, and learning such as the orbitofrontal cortex, nucleus accumbens, and amygdala [164]. Projections of the lateral hypothalamus send outputs practically to all brain regions [164]. Thus, inflammation in the arcuate nucleus, as is the case in some obese patients, can influence the input and output signals to other brain regions, affecting, thus, cognition and mood [58] (Figure 2).

Over the last decades, it has been revealed that the brain monoaminergic systems, which include serotonin (5 hydroxy tryptamin 5HT), dopamine, and noradrenaline, play a vital role in the pathophysiology of depression and many useful antidepressant drugs act via these systems. It has been described for many years now that cytokines can influence monoamine secretion, affecting, thus, neurotransmission and behavior. More specifically, results in animal studies support the concept that IL-1β can act directly to anterior hypothalamus and trigger secretion of norepinephrine, dopamine, and 5 HT [165]. Moreover, it has been proposed that IL-2 administration inhibits electrical stimulation from lateral hypothalamus, decreases dopamine release from nucleus accumbens, while IL-6 can decrease dopamine levels and increase 5-HIAA, an important serotonin metabolite [166]. In addition, IL-1 and IL-6 when affected by stressors can cause further monoamine alterations [166]. In line with these results, recent meta-analyses indicate that anti-inflammatory factors, such as NSAIDs and cytokine inhibitors, can be safely used in the treatment of major depressive disorders or symptoms [167,168]. According to these results, behavioral alterations such as depressive symptoms can be associated with the proinflammatory cytokine profile in the hypothalamic and other regions of the central nervous system.

The interaction between the HPA axis and the monoaminergic systems has been studied for many years now [169]. In particular, results from animal studies showed that 5-HT1A and PT2A serotonin receptors are present in the paraventricular nucleus and that the administration of a 5HT1A agonist intervenes with ACTH secretion [170,171,172]. These data indicate that an altered serotoninergic system can influence the HPA axis. Moreover, it has been hypothesized that systemic inflammation and augmented glucocorticoid levels can decrease serotonin availability in the brain, by changing the tryptophane catabolism pathway in favor of the production of kynurine rather than 5HT [173,174]. Dopamine receptors are also present in the paraventricular nucleus, and elevated glucocorticoids, as in stress conditions, can trigger dopamine release [175,176]. Also, the sensitivity of noradrenaline receptors is influenced by circulating glucocorticoids and CRH can increase noradrenaline release [177], suggesting the interplay between HPA axis and the locus ceruleus/noradrenalinergic system. In line with these, it is claimed that antidepressant drugs regulate cytokines function through, among others, the HPA axis [178].

Along with the monoaminergic systems, the neurotransmitters GABA (gamma-amino butyric acid) and glutamate acid contribute in the pathophysiology of depression [179]. There is also an interplay between these neurotransmitters and the HPA axis. According to animal studies, the administration of glutamate acid in the paraventricular nucleus triggers CRH release and increases ACTH and glucocorticoids levels [179]. At the same time, it is believed that GABA can inhibit the HPA axis through its action to parvocellular neurons in the paraventricular nucleus, preventing its hyperactivation [180].

In addition, the impact of elevated glucocorticoids in memory has been well studied [129]. More specifically, two types of GRs have been identified in the central nervous system: Receptors type I (mineralocorticoid receptor) and receptors type II [181]. Receptors type I are expressed mostly in the hippocampus and other regions of the limbic system and are linked to memory improvement, whereas the type II receptors are expressed in the hypothalamus, hippocampus, and cortical regions and are linked to memory deterioration [129,181]. Chronically elevated glucocorticoid levels favor the expression of type II rather than type I receptors, causing reduction of hippocampal neurogenesis and memory impairment [182]. According to research results, increased glucocorticoids levels can impair cognition. For example, a deterioration of declarative memory following a four-day dexamethasone administration has been reported [183]. Moreover, acute glucocorticoid administration in male students was found to decrease autobiographic memory [184]. Animal studies also support that stress and elevated glucocorticoids can attenuate spatial memory [185,186]. Chronic exposure to high levels of glucocorticoids, due to aging or diseases such as Cushing syndrome and major depressive disorder, can provoke hippocampal atrophy [182,187,188]. Hippocampus is interconnected with the hypothalamus and normally restrains the HPA axis. Hippocampal atrophy impairs this inhibitory action, leading to high levels of glucocorticoids that, in turn, provoke further hippocampal damage, creating, thus, a vicious circle [182]. In addition, glucocorticoids levels have been associated with AD, through many mechanisms. For example, glucocorticoids can augment amyloid beta and tau levels [189]. Moreover, glucocorticoids can trigger enzymes that take part in amyloid beta production and influence the expression of genes implicated in the pathophysiology of AD [190,191]. In addition, a recent animal study indicated that high-fat diet-induced obesity can cause IL-1-mediated activation of microglia in the hippocampus, impacting memory [192]. Another animal study showed that there is astrogliosis in paraventricular and arcuate nucleus of hypothalamus and in hippocampus of obese rats and that this fact is probably associated with memory deficits in these rats [193]. A recent human study used MRI imaging to investigate if brain structure alterations in obese patients compared to normal weight controls are similar with those in patients with neuropsychiatric disorders [194]. The results support the notion that obesity affects cortical thickness in a similar way that major depression does, as previous studies reported [194,195]. Another recent cross-sectional study used diffusion tensor imaging, an MRI technique, to correlate hypothalamic damage in middle-aged obese individuals with cognitive defects. More specifically, along with the MRI imaging, cognitive performance was assessed with specific tests and was compared to the normal-weight control group. The results indicated that there is an association between obesity-induced hypothalamic injury and worse cognitive performance [196]. The evidence provided was not strong because of the small sample studied, so more human studies on the topic are needed.

## 6. Potential Prevention of Mood and Cognitive Disorders by Treating Hypothalamic Inflammation

As discussed above, it is well established that obesity can induce hypothalamic inflammation. Consequently, recent research has focused on potential treatment of obesity-induced hypothalamic inflammation. According to animal studies, exercise, such as running, seems to reduce diet-induced hypothalamic inflammation in rodents [197]. In line with these results, swimming and diet can diminish not only hypothalamic inflammation, but also memory decline in an APOE4 mice model [198]. In addition, dietary options can improve hypothalamic inflammation. Unsaturated fatty acids can decrease diet-induced hypothalamic inflammation in mouse models [199]. Other dietary substances such as green tea polyphenol have been proposed as factors that reduce hypothalamic inflammation in obese models [200]. Furthermore, glucagon-like peptide 1 (GLP-1) receptor agonists are widely used for the treatment of obesity and DM. As GLP-1 receptors exist in the hypothalamic arcuate nucleus, GLP-1 receptor agonists can act in the arcuate nucleus and impact appetite [201]. Interestingly, recent studies support that GLP-1 receptor agonists have a neuroprotective role and can decrease hypothalamic inflammation [202]. In an animal study, liraglutide can improve cognitive decline in rodents with AD, revealing, thus, a new approach to the treatment of neurodegenerative diseases [203].

Taking into consideration the aforementioned data which support that hypothalamic inflammation may be implicated in the pathogenesis of mood and cognitive disorders, it can be hypothesized that diet, exercise, and GLP-1 receptor agonists could also improve these morbidities in obese patients. Future research could investigate the efficacy of these strategies in obese patients with neuropsychiatric co-morbidities, in order to further ameliorate the therapeutic options of these patients.

## 7. Conclusions

As discussed above, epidemiological data provide evidence that obesity often occurs in association with mood and cognitive disorders. It is well established that along with the systematic inflammation, obesity is associated with neuroinflammation. Obesity induces inflammation not only in hypothalamus, but also in other brain areas, such as the hippocampus, affecting, thus, mood and memory. The interconnection of the hypothalamus with many brain areas, such as hippocampus, amygdala, and cortical areas, can explain the impact of the hypothalamus on cognition and mood. In addition, increased levels of inflammatory cytokines can affect the function of the HPA axis, which plays an important role in cognition and mood. Taking into consideration all of the above, diet-induced hypothalamic inflammation is associated with cognitive decline and depression. Despite the common pathophysiological mechanisms shared between obesity, cognitive, and mood disorders, there are not yet enough studies to claim that improvement or reversal of hypothalamic inflammation could prevent cognitive and mood disorders or delay the cognitive decline in the early stages of neurodegenerative diseases. Further research is needed towards this direction. Most of the studies concerning the topic are animal studies that investigate the possible pathways between obesity and neuropsychiatric disorders. Most of the available human studies include epidemiological data which indicate the relationship between obesity and cognitive disorders or depression, but only one human study with a small sample of participants exists associating directly hypothalamic inflammation in obese subjects with these disorders. More studies need to focus on brain regions that may be affected by diet/obesity-induced inflammation and their association with cognitive and mood disorders. Detailed knowledge of common pathophysiological pathways will reveal new approaches for prevention and therapy of these morbidities. For example, possible prevention of cognitive decline could be a strong motivation for lifestyle alteration aiming at lowering body weight. Recent studies support that exercise can anticipate high-fat induced neuroinflammation [197] and that a Mediterranean diet is linked with reduced brain atrophy [192]. Future investigation is needed to highlight the type of micro- or macro- nutrients which can alter the process of hypothalamic inflammation and prevent cognitive impairment and mood disorders.

## Figures and Tables

**Table 1:** Proposed mechanisms leading to obesity-induced hypothalamic inflammation.

Stimuli of Inflammatory Mechanisms	Mechanisms
Saturated fatty acids	Activation of TLR-4 pathway, Increased proinflammatory cytokines, Microglia activation
Saturated fatty acids, Hyperlipidemia Hyperglycemia	ER stress response
Western diet	Increased BBB permeability
High-fat diet	Disrupted autophagy

## Data Availability

Not applicable.
